# Dr. Eliot on the Relation of Sex Education to Eugenics

**Published:** 1914-07

**Authors:** 


**Dr. Eliot on the Relation of Sex Education to Eugenics.**

---

Dr. Eliot, President-elect of the American Social Hygiene Association, in a recent address (Public Opinion and Sex Hygiene), very pointedly stated the following position:

"The interest of many thinking people in the subject of eugenics is closely allied in interest in sex hygiene. * * * Public progress in relation to sex hygiene and eugenics is to be procured chiefly through educational methods. It is therefore of the utmost importance that the processes adopted for diffusing sound knowledge should all be carried on plainly but delicately, without exaggeration or morbid suggestion, without interference with parental rights or religious convictions, and in general in a pure, high-minded, disinterested way. The pioneering part of this work must be done by voluntary associations, as is usual in social reforms; but it should be the constant aim of these private organizations to enlist gradually the public authorities in this vast undertaking, and to transfer to the public treasury as fast as possible the support of all those parts of the work which experience proves to be of sure and permanent public advantage."

---

PROSPECTIVE.—The medical journal of the future is not to be the official journal which is without independence, nor yet the independent journal which offers no official service.

The journal regarded with respect, and valued by its subscribers, is the independent journal which is the servant of the societies whose members are its readers and supporters.

The hope that an official society journal may be independent is remote. That independent journals may become officially most useful servants is no longer a hope but a fact.

The definite progress of the past year has been along this line, and it is definitely along this line that the progress of the New Year will be made.—*Boston Medical and Surgical Journal.*

---

AFTER ALL, the kind of world one carries about in one's self is the important thing, and the world outside takes its grace, color and value from that.—*Lowell.*

---

THE BEDBUG SPREADS TUBERCULOSIS, according to Dr. J. Walling Beveridge, of New York. The evidence is accumulating that it is an insect as dangerous to human life as the fly or Dum-Dum mosquito. It has been known for some time that it is responsible for the spread of fever, and recently it has been charged with the causation of leprosy and epidemic spinal meningitis. That it may also serve as a carrier for tuberculosis seems extremely likely. The fact that the bedbug may live for six months or more without food makes it especially hard to get rid of, and naturally increases the danger to a considerable extent.—*Ex.*

